# Diagnostic utility of chest wall vessel involvement sign on ultra-high-resolution CT for primary lung cancer infiltrating the chest wall

**DOI:** 10.1007/s00330-025-11382-x

**Published:** 2025-01-28

**Authors:** Fuga Uota, Shingo Iwano, Shinichiro Kamiya, Rintaro Ito, Shota Nakamura, Toyofumi Fengshi Chen-Yoshikawa, Shinji Naganawa

**Affiliations:** 1 Department of Radiology, Nagoya University Graduate School of Medicine, 65 Tsurumai-cho, Showa-ku, Nagoya, 466-8550 Japan; 2 Department of Thoracic Surgery, Nagoya University Graduate School of Medicine, 65 Tsurumai-cho, Showa-ku, Nagoya, 466-8550 Japan

**Keywords:** Carcinoma (Non-small-cell lung), neoplasm staging, Pleura, Computed tomography Angiography, Imaging (Three-dimensional)

## Abstract

**Objectives:**

Chest wall infiltration in primary lung cancer affects the surgical and therapeutic strategies. This study evaluates the efficacy of the chest wall vessel involvement in subpleural lung cancer (CWVI)　on ultra-high-resolution CT (UHR-CT) for detecting chest wall invasion.

**Materials and methods:**

A retrospective analysis of lung cancer cases with confirmed pleural and chest wall invasion was conducted from November 2019 to April 2022*.* Seventy-seven patients (mean ± standard deviation age 70 ± 8 years, 64 males) who underwent preoperative contrast-enhanced UHR-CT were included. They were grouped into 51 non-chest wall infiltration (pl1 and pl2) and 26 chest wall infiltration (pl3). Clinical, histopathological, and UHR-CT findings were reviewed.

**Results:**

Upper lobe tumors exhibited a higher chest wall invasion rate (*p* < 0.001). Rib destruction was evident in five patients with chest wall invasion but none with pleural invasion (*p* < 0.001). CWVI was present in 19 of 26 patients with chest wall invasion and 2 of 51 patients with pleural invasion (*p* < 0.001). The maximum tumor diameter (Dmax), arch distance which means the interface length between the primary tumor and the chest wall (Adist), and the ratio of Dmax to Adist were higher in chest wall invasion cases (all *p* < 0.001). After excluding patients with rib destruction, in multivariate logistic regression analysis, only CWVI was a significant predictor for chest wall invasion (odds ratio 29.22 (95% confidence interval 9.13–262.90), *p* < 0.001).

**Conclusion:**

CWVI on UHR-CT can help diagnose lung cancer infiltrating the chest wall, offering a potential tool for clinical decision-making.

**Key Points:**

***Question*** *Chest wall infiltration in primary lung cancer has implications for the treatment plan, but diagnosis is often difficult with conventional CT.*

***Findings*** *Chest wall vessel involvement in subpleural lung cancer on ultra-high-resolution CT is a valuable predictor for diagnosing chest wall infiltration.*

***Clinical relevance*** *The delineation of chest wall vessels with contrast-enhanced ultra-high-resolution CT may improve the diagnosis of chest wall infiltration and allow accurate staging and optimal treatment options for subpleural primary lung cancer.*

**Graphical Abstract:**

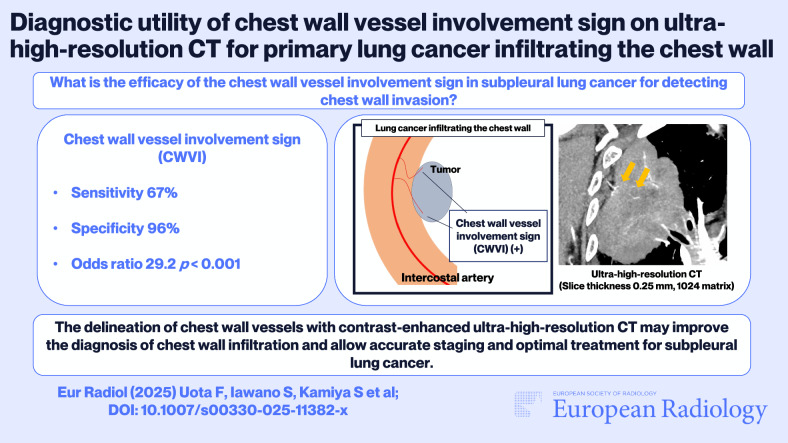

## Introduction

Chest wall infiltration of lung cancer corresponds to T3 in the TNM classification 8th edition by the Union for International Cancer Control (UICC). It greatly influences the therapeutic strategy and prognosis for primary lung cancer [1–4]. For complete resection of lung cancer infiltrating the chest wall, en bloc lung and chest wall resections are required; therefore, the preoperative imaging of resectable lung cancer infiltrating the chest wall is crucial. The extent of chest wall infiltration can be classified as parietal pleural, endothoracic fascial, or rib. Diagnosing rib destruction is relatively straightforward using CT or MRI, whereas diagnosing parietal pleural and endothoracic fascial invasion is often complex.

Classic CT findings of chest wall infiltration are based on rib destruction, tumor diameter, and contact length or angle between the tumor and the chest wall. Diagnosing chest wall infiltration by tumor movement using respiratory dynamic CT and cine MRI has also been reported [5–14].

In our practice, we noticed the presence of vessels spreading from the intercostal artery into the tumor, and we have termed them the “chest wall vessel involvement in subpleural lung cancer (CWVI).” Although the main feeding artery for primary lung cancer is the bronchial artery, it has been previously reported that lung cancers receive blood from an intercostal artery on angiography [15–17]. Therefore, we hypothesized that lung cancer that infiltrates the chest wall may generate feeding arteries from the arteries of the chest wall. We suppose it is possible to diagnose the invasion of arteries distributed in the chest wall, such as the intercostal arteries, within the tumor using contrast-enhanced CT. In that case, chest wall infiltration may be diagnosed. However, invasive angiography is rarely performed to analyze the feeding vessels. Instead, we have attempted to use ultra-high-resolution CT (UHR-CT), which has recently become available for practical applications [18–21] to detect minute CWVI. The UHR-CT differs from conventional CT systems by using smaller detector elements (0.25 × 0.25 mm) and an X-ray tube with a smaller focal spot (0.4 × 0.5 mm), allowing for higher spatial resolution images [21]. Additionally, the UHR-CT systems support matrix sizes ranging from 1024 × 1024 to 2048 × 2048, further enhancing image clarity and detail. Furthermore, it has been reported that image quality can be improved using a deep-learning-based reconstruction algorithm for UHR-CT image reconstruction [22–24].

Herein, we examined the frequency of CWVI in lung cancer with chest wall infiltration on UHR-CT and the diagnostic suitability of CWVI for chest wall infiltration.

## Methods

Our institutional review board approved this retrospective study and waived the requirement for written informed consent (approval no.: 2022-0162).

### Patient selection

The UHR-CT was installed at our hospital at the end of 2019 and began scanning for lung cancer in 2020. Therefore, we searched for postoperative histopathology reports of primary lung cancer cases operated on at our institution between January 2020 and April 2022 to identify pathologically proven pleural invasion and chest wall infiltration. Inclusion criteria were postoperative histopathological evidence of invasion of the visceral pleura or greater (pl1 as invasion beyond the elastic layer, pl2 as invasion to the surface of the visceral pleura, and pl3 as invasion of the chest wall, diaphragm, mediastinum or adjacent lung lobe). Exclusion criteria were the absence of chest wall-tumor contact on CT and the lack of UHR-CT data. Consequently, tumors involving the diaphragm, mediastinum, or interlobar pl3 were excluded. Selected lung cancers classified as pl1 and pl2 were categorized as the non-chest wall infiltration group, while those classified as pl3 were categorized as the chest wall infiltration group. We recorded patient characteristics and symptoms (age, sex, body weight, and chest pain) from medical records and tumor characteristics (lung lobe in the existence of a tumor, pathological tumor size, histological type, and TNM staging) from surgical and pathological records. TNM staging system (UICC, 8th edition) was used.

### CT scan protocol

All CT scans were performed using the super-high-resolution mode of a UHR-CT scanner (Aquilion Precision; Canon Medical Systems Corp.) in the craniocaudal direction during inspiratory apnea, with dynamic contrast enhancement. The following scan parameters were used: beam collimation, 0.25 mm × 160 rows; x-ray tube voltage, 120 kV; tube current, auto exposure control (Standard Deviation 20); rotation speed, 0.5 s; helical pitch, 0.806.

At our hospital, two-phase (arterial phase and venous phase) dynamic CT scans are routinely performed for preoperative lung cancer, and we used arterial phase scan image data in the present study. The iodine contrast agent (96 mL) was administered intravenously at a rate of 4 mL/s, followed by 20 mL of saline at 4 mL/s. The iodine dosage was 240 mg/mL for patients weighing < 45 kg, 320 mg/mL for patients weighing 45–55 kg, and 370 mg/mL for patients weighing ≥ 55 kg. The bolus tracking method was used for the arterial phase, and the scan was initiated when the ROI of the ascending aorta exceeded 120 HU.

The UHR-CT images were reconstructed with a matrix of 1024 × 1024, field of view of 320 mm, slice thickness of 0.25 mm, slice interval of 0.25 mm, and deep-learning reconstruction kernel (AiCE Body_sharp Standard).

The reconstructed image data were transferred to the picture archiving and communication system (PACS) in our hospital and were displayed at window settings for lung (level −600 HU; width, 1600 HU) and soft tissue (level, 25 HU; width, 330 HU).

### CT assessment

**Fig. 1:**
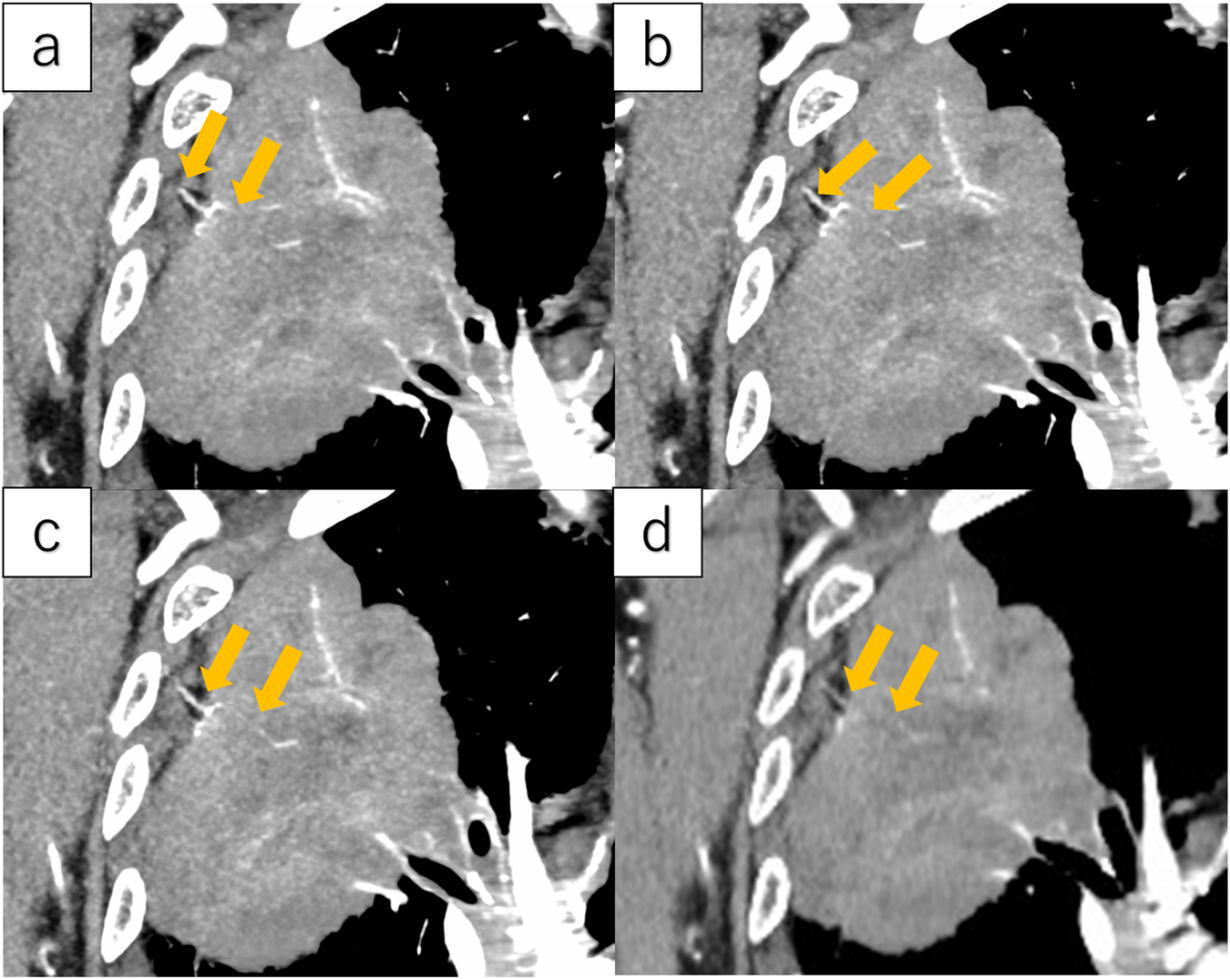
Maximum intensity projection created from ultra-high-resolution CT images Ultra-high-resolution CT images of a 79-year-old female showing adenocarcinoma infiltrating the chest wall in the right upper lobe. **a**, **b**, and **c** are coronal sections arranged ventral to dorsal. **d** is a conventional HRCT image. The tumor diameter was 85 mm and showed chest wall vessel involvement in subpleural lung cancer (arrow). The postoperative pathology assessment confirmed parietal pleural invasion of the tumor

**Fig. 2:**
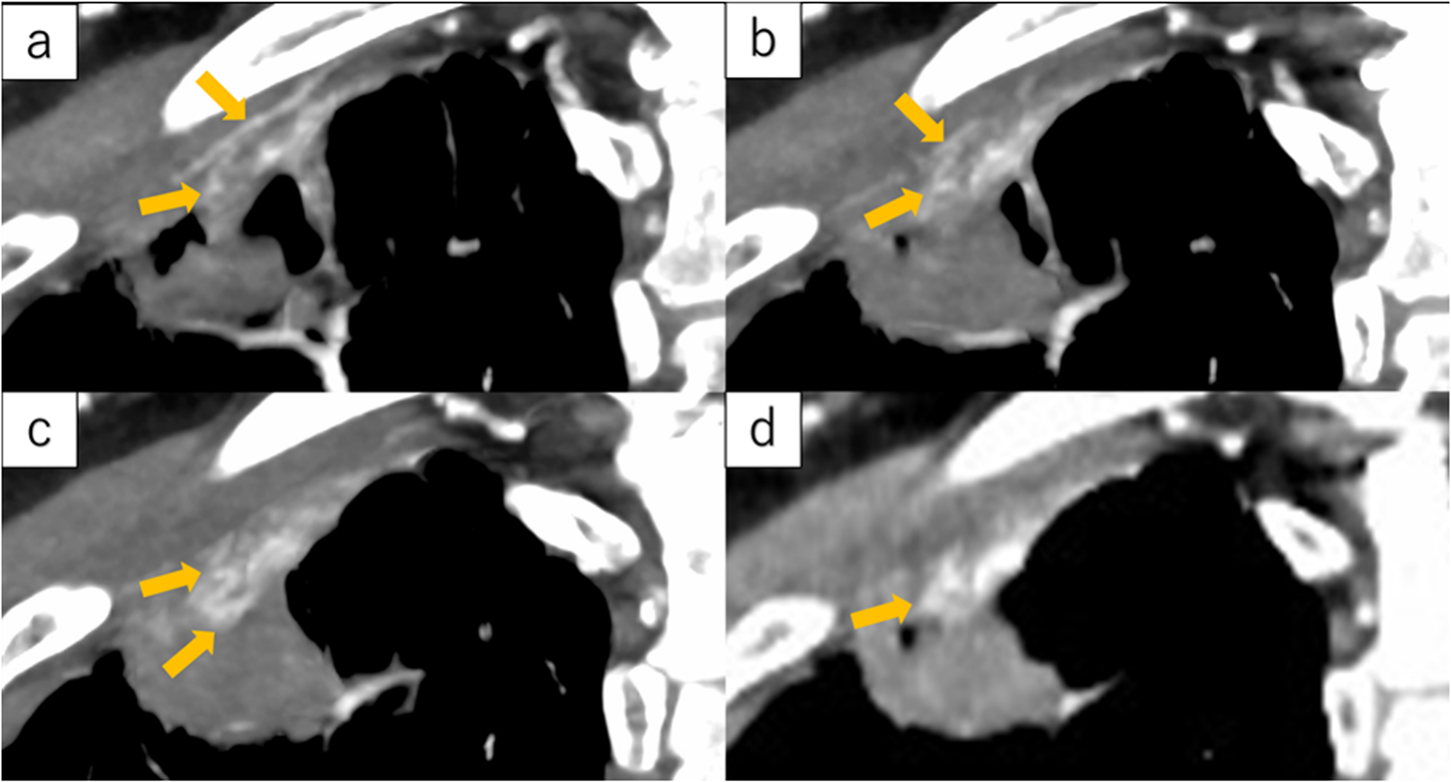
Maximum intensity projection created from ultra-high-resolution CT images of a 53-year-old female showing adenocarcinoma infiltrating the chest wall in the right upper lobe. **a**, **b**, and **c** are coronal sections arranged ventral to dorsal. **d** is a conventional HRCT image. The tumor diameter was 24 mm, and showed chest wall vessel involvement in subpleural lung cancer (arrow). The postoperative pathology assessment confirmed parietal pleural invasion of the tumor

**Fig. 3:**
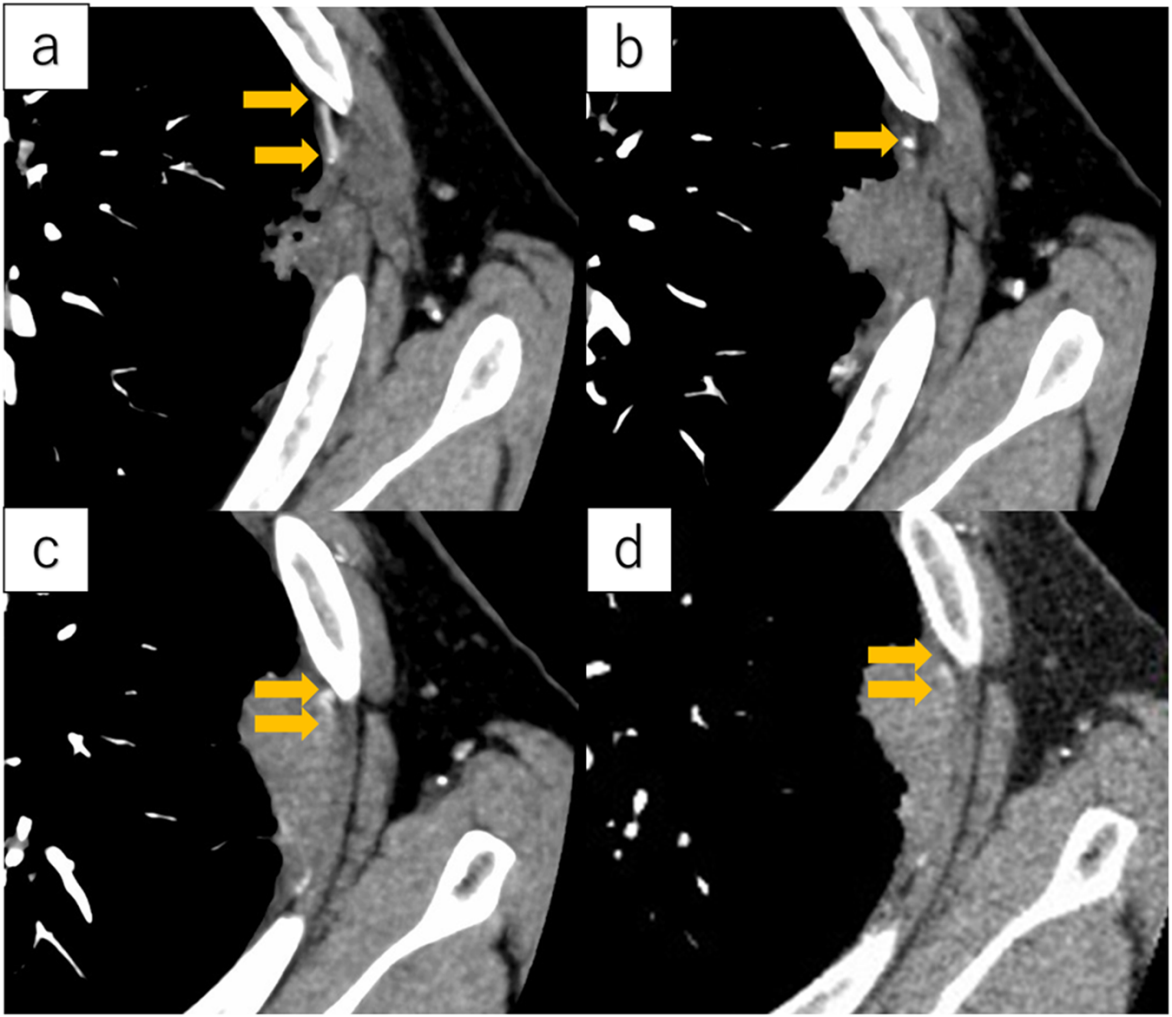
Ultra-high-resolution CT images of a 73-year-old male showing squamous cell carcinoma infiltrating the chest wall in the left upper lobe. **a**, **b**, and **c** are axial sections arranged from bottom to top. **d** is a conventional HRCT image. The tumor diameter was 29 mm and showed chest wall vessel involvement in subpleural lung cancer (arrow). The postoperative pathology assessment confirmed parietal pleural invasion of the tumor

Two observers (a thoracic radiologist with 29 years of reading experience and a radiological resident with three years of reading experience) independently reviewed the UHR-CT images. They were blinded to the postoperative pathological diagnosis. Before the actual reading, findings were assessed using training UHR-CT images of 10 non-participating individuals (5 patients with chest wall infiltration and five patients without chest wall infiltration). They assessed the presence or absence of CWVI, rib destruction, pleural effusion, and ground-glass opacity (GGO) in the tumor. CWVI and rib destruction were rated as positive, equivocal, or negative. CWVI was evaluated as positive when vessels distributed from the intercostal artery to the tumor could be identified. Figures 1–3 show the CWVI-positive images. Pleural effusion and GGO were rated as positive or negative, respectively. When different findings were reported between the two observers, the decision was finalized by consensus.

The maximum tumor diameter (Dmax) and the arch distance to the chest wall (Adist) were measured on an axial section using the PACS measurement tool, and the Adist-to-Dmax ratio (the ratio of maximum tumor diameter to arch distance with the chest wall (A/D ratio)) was calculated [13, 25]. Arch distance means the interface length between the primary tumor and the chest wall. Dmax and Adist were measured three times to reduce subjectivity, and the average values were used.

### Statistical analysis

First, the inter-reader reliability between the two readers was analyzed using Cohen’s kappa coefficient (k). The degree of agreement was interpreted as follows: less than 0.20, poor agreement; 0.21–0.40, fair agreement; 0.41–0.60, moderate agreement; 0.61–0.80, substantial agreement; and 0.81–1.00, almost perfect agreement.

Next, the selected tumors were classified into two groups: lung cancers not infiltrating the chest wall (pl1 and pl2) and lung cancers infiltrating the chest wall (pl3), and the two groups were compared. The age, pathological tumor size, Dmax, Adist, and A/D ratio were compared with t-tests, and the frequency of sex, histological type, lobe, pleural effusion, rib destruction, and CWVI were compared with the *χ*^2^-test. The exact comparisons were performed for patients in whom no rib destruction was observed.

Finally, univariate and multivariate logistic regression analyses were performed to determine the significance of chest wall infiltration in patients whose rib destruction was not observed. Significant variables in the univariate analysis were adopted for multivariate analysis, and a stepwise method was used for further variable selection.

Analyses were performed using SPSS version 28 (IBM Corp.), Excel 2019 (Microsoft Corp.), with an add-in statistical software BellCurve for Excel version 4.04 (Social Survey Research Information Corp.). Statistical significance was set at *p* < 0.05.

## Results

**Fig. 4:**
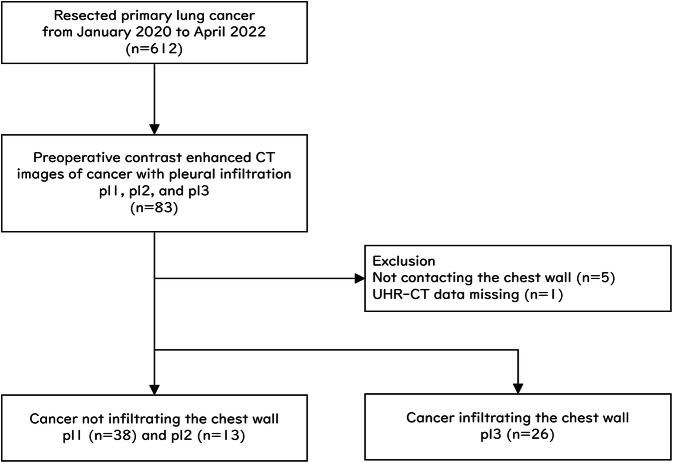
Study cohort flowchart. After applying the exclusion criteria, 77 patients with lung cancers were included in the present analysis

From January 2020 to April 2022, 612 primary lung cancer surgeries were performed at our institution. Patients with proven non-chest wall infiltration (pl1 and pl2) and chest wall infiltration (pl3) were selected for postoperative histopathology (*n* = 83) (Fig. 4). Cases in which chest wall infiltration was suspected preoperatively but could not be proven due to complete tumor resolution by preoperative neoadjuvant therapy were not included. Patients without chest wall contact on CT (*n* = 5) and with missing UHR-CT data (*n* = 1) were excluded, and patients (*n* = 77, 13 females and 64 males; mean ± standard deviation (SD) age 70 ± 8 years; mean ± SD body weight 60.4 ± 10.2 kg) were included. For example, cases with tumor invasion into the interlobar pleura or mediastinal pleura but not into the chest wall were excluded. Of these patients, 51 had cancers not infiltrating the chest wall, and 26 had cancers infiltrating the chest walls. The histological type in 47 patients was adenocarcinomas, in 24 was squamous cell carcinomas, in 3 was large cell carcinomas, in 2 was adenosquamous carcinomas, and in 1 was small cell carcinoma. Six patients received neoadjuvant therapy. The pathological TNM stage was stage I in 30 patients, stage II in 27, stage III in 17, and stage IV in 3. In 71 patients without neoadjuvant therapy, the mean time from UHR-CT to surgery was 25 ± 12 days. Conversely, in the six patients treated with neoadjuvant therapy, the mean time from UHR-CT to surgery was 73 ± 45 days. The mean CTDIvol and DLP of UHR-CT were 11.5 ± 2.8 mGy and 473.2 ± 116.6 mGy･cm. The mean scan delay time after contrast medium injection start was 22.2 ± 2.1 s.

Regarding the mean ± SD pathological tumor size, the Dmax and Adist were 3.8 ± 2.0 cm, 3.8 ± 1.9 cm, and 3.7 ± 3.2 cm, respectively. Rib destruction and CWVI were observed in 5 and 21 patients, respectively. For the rib destruction and CWVI, there was strong agreement between observers (*κ* = 0.69 and *k* = 0.77, respectively). For pleural effusion and GGO, the agreement between the observers was fair (*k* = 0.47 and *k* = 0.55, respectively).

A comparison of the chest wall infiltrating and non-infiltrating groups showed that chest pain, tumors in the upper lobes, rib destruction, and CWVI frequencies were significantly higher in the infiltrating group (*p* = 0.030, *p* < 0.001, *p* = 0.003, and *p* < 0.001, respectively; Table 1). The frequencies of CWVI on the UHR-CT images were 73% (19/26) and 4% (2/51) in patients with and without chest wall infiltration, respectively. The sensitivity, specificity, positive predictive value (PPV), and negative predictive value of CWVI for diagnosing chest wall infiltration were 73% (19/26), 96% (49/51), 90% (19/21), and 88% (49/56), respectively. The Dmax, Adist, and A/D ratios were also significantly higher in the infiltrating group (all *p* < 0.001). The mean age was significantly lower in the infiltrating group (*p* = 0.048); however, no significant differences were observed in sex, histopathology, pleural effusion, or GGO within the tumor.

**Table 1:** Comparison of patient and tumor characteristics between the non-infiltrating and infiltrating chest wall groups

	Non-infiltrating	Infiltrating	*p*-value
Number (*n*)	51	26	
Age (years)	71 ± 8	67 ± 6	0.048
Male/Female (*n*)	41/10	23/3	0.525
Chest pain −/+ (*n*)	51/0	18/8	0.030
Adenocarcinoma/others (*n*)	32/19	15/11	0.667
Upper lobe/others (*n*)	21/30	21/5	< 0.001
Pathological Size (cm)	3.5 ± 1.7	4.3 ± 2.4	0.172
Dmax (cm)	3.5 ± 1.6	4.8 ± 2.0	< 0.001
Adist (cm)	2.6 ± 1.9	6.0 ± 4.2	< 0.001
A/D ratio	0.76 ± 0.30	1.16 ± 0.38	< 0.001
Pleural Effusion −/+ (*n*)	36/15	16/10	0.423
GGO −/+ (n)	41/10	24/2	0.318
Rib Destruction −/+ (*n*)	51/0	21/5	0.003
CWVI −/+ (*n*)	49/2	7/19	< 0.001

*CWVI* chest wall vessel involvement in subpleural lung cancer, *Dmax* maximum tumor diameter, *Adist* arch distance with the chest wall, *A/D ratio* ratio of Adist to Dmax, *GGO* ground-glass opacity

After excluding five patients with rib destruction, tumors in the upper lobe and CWVI were significantly more frequent in the infiltrating group (*p* = 0.009 and *p* < 0.001, respectively; Table 2). In this sub-group, the frequencies of CWVI were 67% (14/21) and 4% (2/51) in patients with and without chest wall infiltration, respectively. The sensitivity, specificity, PPV, and NPV of CWVI for diagnosing chest wall infiltration were 67% (14/21), 96% (49/51), 88% (14/16), and 88% (49/56), respectively. Dmax, Adist, and A/D ratios were significantly higher in the infiltrating group (*p* = 0.007, *p* < 0.001, and *p* < 0.001, respectively), and no significant differences were observed in age, sex, chest pain, histopathology, pleural effusion, or GGO within the tumors.

**Table 2:** Comparison of patient and tumor characteristics between the non-infiltrating and infiltrating chest wall groups except for rib-destructive tumors

	Non-infiltrating	Infiltrating	*p*-value
Number (*n*)	51	21	
Age (years)	71 ± 8	68 ± 6	0.182
Male/Female (*n*)	41/10	18/3	0.743
Chest pain −/+ (*n*)	51/0	18/3	0.083
Adenocarcinoma/others (*n*)	32/19	13/8	0.947
Upper lobe/others (*n*)	21/30	16/5	0.009
Pathological size (cm)	3.5 ± 1.7	4.2 ± 2.2	0.187
Dmax (cm)	3.3 ± 1.6	4.5 ± 1.7	0.007
Adist (cm)	2.6 ± 1.9	4.9 ± 2.7	< 0.001
A/D ratio	0.76 ± 0.30	1.07 ± 0.32	< 0.001
Pleural effusion −/+ (*n*)	36/15	14/7	0.743
GGO −/+ (*n*)	41/10	19/2	0.489
Rib destruction −/+ (*n*)	51/0	21/0	N/A
CWVI −/+ (*n*)	49/2	7/14	< 0.001

*CWVI* chest wall vessel involvement in subpleural lung cancer, Dmax maximum tumor diameter, *Adist* arch distance with chest wall, *A/D ratio* ratio of Adist to Dmax, *GGO* ground-glass opacity

In the univariate logistic regression analysis for the diagnosis of chest wall infiltration, tumors in the upper lobe (*p* = 0.010), Dmax (*p* = 0.014), Adist (*p* = 0.001), A/D ratio (*p* = 0.002), and CWVI (*p* < 0.001) were significant factors in 72 patients without rib destruction. The multivariate analysis revealed that CWVI (*p* < 0.001) was the only significant predictor of chest wall infiltration (Table 3). Chest pain was not an appropriate factor for logistic analysis because it was only seen in the infiltrating group.

**Table 3:** Result of the univariate and multivariate logistic regression analyses

	Univariate			Multivariate		
	OR	95% CI	*p*-value	OR	95% CI	*p*-value
Age	0.96	0.90–1.02	0.183			
Male/Female	1.46	0.36–5.96	0.595			
Adenocarcinoma	0.97	0.34–2.75	0.947			
Upper lobe	4.57	1.45–14.42	0.010	4.15	0.87–19.75	0.074
Dmax	1.04	1.01–1.08	0.014			
Adist	1.05	1.02–1.08	0.001			
A/D ratio	40.77	4.04–411.56	0.002	9.77	0.72–132.32	0.086
Pleural effusion	1.20	0.40–3.57	0.743			
GGO	0.43	0.09–2.17	0.307			
CWVI	49.00	9.13–262.90	< 0.001	29.22	4.83–176.86	< 0.001

*CWVI* chest wall vessel involvement in subpleural lung cancer, *Dmax* maximum tumor diameter, *Adist* arch distance with chest wall, *A/D ratio* ratio of Adist to Dmax, *GGO* ground-glass opacity, *CI* confidence interval, *OR* odds ratio

## Discussion

Herein, we investigated the ability of CWVI to diagnose chest wall infiltration in patients with primary lung cancer using contrast-enhanced UHR-CT. CWVI was observed in 19 of 26 (73%) cancers infiltrating the chest wall, with good agreement between observers. Without rib destruction, CWVI was also observed in 14 of 21 (67%) cancers infiltrating the chest wall. In the univariate logistic regression analysis, tumors in the upper lobe, Dmax, Adist, A/D ratio, and CWVI were significant factors in the diagnosis of cancers infiltrating the chest wall. CWVI was a significant predictor in the multivariate logistic analysis. We observed CWVI in 19 of 26 (73%) patients with cancers infiltrating the chest wall. There are two possible reasons why CWVI is more common in patients with cancer infiltrating the chest wall. One possibility is that the tumor may have infiltrated the preexisting intercostal arteries in the chest wall. Another possibility is that the tumor that infiltrated the chest wall may have induced angiogenesis in the arteries of the chest wall. It has been previously reported that lung cancers infiltrating the chest wall receive blood supply from the intercostal artery on angiography [15–17]. Angiogenesis from the arteries in the chest wall may result from vascular endothelial growth factor (VEGF) secreted by the tumor. Cancer infiltrating the chest wall can also cause inflammation of the surrounding tissues, which may contribute to angiogenesis. When chronic inflammatory diseases such as pulmonary tuberculosis and aspergillosis come into contact with the pleura, various non-bronchial artery-derived vessels can develop through the adherent pleura [26].

Our institution routinely uses diagnostic CT angiography with 1-mm-thickness, 512-matrix reconstructed images. In this study, we examined 0.25-mm-thickness, 1024-matrix reconstructed images to delineate thin vessels that cannot be delineated with routine CT images. In particular, the resolution was four-fold higher in the *z*-axis direction, which resulted in improved resolution of the MPR sagittal and coronal section images. Because lung cancer contacts the chest wall in various directions at the apex of the lung, the detection ability of CWVI improved by observing the MPR images in three dimensions. Rib destruction by tumors is beneficial for diagnosing chest wall infiltration. In our study, all five patients with rib destruction were pathologically proven to have cancers infiltrating the chest wall. However, when tumor infiltration is limited to the parietal pleura or endothoracic fascia and rib destruction is not evident, diagnosing chest wall infiltration using CT becomes challenging. Therefore, we limited our comparison to patients without rib destruction and used logistic regression analysis to examine other imaging features helpful in diagnosing chest wall infiltration. The results showed that the upper lobe, Dmax, Adist, A/D ratio, and CWVI were significant predictors of chest wall infiltration. In our study, lung cancers in the upper lobes showed a higher frequency of chest wall infiltration than those in the middle and lower lobes. One possible reason may be that the upper lobe undergoes less lung movement than the middle and lower lobes during respiration. The middle and lower lobes are deformed to a greater extent than the upper lobe owing to the movement of the diaphragm, in addition to the movement of the thorax [27], and the time and extent of contact between the tumor and parietal pleura is limited. Conversely, the upper lobe is prone to adhesion between the visceral and parietal pleura due to old inflammation, such as pulmonary tuberculosis, and lung cancer is likely to infiltrate the chest wall. Dmax and Adist are factors that relate to tumor size. The larger the tumor, the greater the area of contact between the tumor and the chest wall, and the higher the risk of chest wall infiltration. Previously, Imai et al reported that tumors with a high A/D ratio (i.e., tumors with a large Adist relative to Dmax) were more likely to infiltrate the chest wall [13]. Dmax was a significant predictor of chest wall infiltration in our study in the univariate logistic regression analysis. We expect the A/D ratio to help diagnose chest wall infiltration in patients who cannot undergo contrast-enhanced CT because of contrast allergy or renal dysfunction.

Lung cancers with chest wall infiltration encompass a range of histologic types, including adenocarcinoma and squamous cell carcinoma, among others [1, 28, 29]. Nevertheless, there was no discernible difference between adenocarcinoma and other histologic types about the infiltration of the cancer in the present study.

This study has a few limitations. First, this was a single-center, retrospective study. The population size was insufficient to confirm the conclusions because operable cancers infiltrating the chest wall are uncommon. Also, patients in whom tumors entirely resolved with preoperative chemoradiotherapy were excluded because pathological evidence of chest wall infiltration could not be obtained, which may have led to a selection bias. The incidence of lung cancer infiltrating the chest wall is low. In our facility, out of 612 primary lung cancer surgeries, 26 cases (4.2%) involved resectable chest wall infiltration. Moving forward, it is essential to verify the effectiveness of CWVI through a multicenter collaborative study focused on its diagnostic accuracy. Second, this retrospective study utilized clinical UHR-CT data from the arterial phase for 3D-CT angiography of the pulmonary arteriovenous system. The average scan start time was 22 s after contrast injection, which appears to be somewhat early for adequate delineation of the intercostal arteries. It is possible that more CWVI could have been detected in lung cancer infiltrating the chest wall if the scan had been taken at the appropriate time to delineate the intercostal arteries. Third, this clinical study was not intended to evaluate UHR-CT image quality, and we did not compare image quality metrics such as contrast-to-noise ratio (CNR) between UHR-CT and conventional HRCT, as several papers have already demonstrated that UHR-CT provides better image quality and clinical diagnostic performance than conventional HRCT. Image quality deterioration due to factors such as obesity and pleural effusion may affect the ability to detect CWVI, although we did not assess these factors. Additionally, we visually diagnosed the presence or absence of nutrient vessels and did not measure vessel diameters. Further research is needed to determine the appropriate image quality for analyzing the presence of CWVI. Finally, the usefulness of contrast-enhanced ultrasound (CEUS) of the chest in diagnosing chest wall infiltration has been reported. Nevertheless, it has not been performed in our institution or considered in this study. UHR-CT combined with CEUS might more accurately diagnose chest wall infiltration [30, 31].

In conclusion, UHR-CT can facilitate the preoperative diagnosis of cancer infiltrating the chest wall. Rib destruction, upper lobe lung cancer, Dmax, Adist, A/D ratio, and CWVI were useful findings to discriminate chest wall infiltration. In particular, CWVI was an independent predictor of chest wall infiltration for cancers without rib destruction.

## Data Availability

Data generated or analyzed during the study are available from the corresponding author by request.

## Abbreviations

A/D ratio: The ratio of maximum tumor diameter to arch distance with the chest wall
Adist: Arch distance with the chest wall
CWVI: Chest wall vessel involvement in subpleural lung cancer
Dmax: Maximum tumor diameter
GGO: Ground-glass opacity
pl1: Invasion beyond the elastic layer
pl2: Invasion to the surface of the visceral pleura
pl3: Invasion of the chest wall, diaphragm, mediastinum or an adjacent lung lobe
UHR-CT: Ultra-high-resolution CT
